# Exploring the relationships between anthropometric indices of adiposity and physical performance in middle-aged and older Brazilian women: a canonical correlation analysis

**DOI:** 10.4178/epih.e2022074

**Published:** 2022-09-13

**Authors:** Rafaela Andrade do Nascimento, Mariana Carmem Apolinário Vieira, Juliana Fernandes, Ingrid Guerra Azevedo, Mayle Andrade Moreira, José Vilton Costa, Saionara Maria Aires da Câmara, Álvaro Campos Cavalcanti Maciel

**Affiliations:** 1 Physiotherapy Department of Federal University of Rio Grande do Norte, Natal, Brazil; 2 Physiotherapy and Collective Health Laboratory, Physiotherapy Department, Federal University of Pernambuco, Recife, Brazil; 3 Departamento de Procesos Terapeuticos, Universidad Católica de Temuco, Temuco, Brazil; 4 Physiotherapy Department of Federal University of Ceará, Rodolfo Teófilo, Brazil; 5 Department of Demography and Actuarial Sciences, Federal University of Rio Grande do Norte, Natal, Brazil

**Keywords:** Aging, Adiposity, Anthropometry

## Abstract

**OBJECTIVES:**

This study analyzed the influence of anthropometric indices of adiposity on the physical performance of middle-aged and older women.

**METHODS:**

A cross-sectional study was conducted among 368 women from 50 years to 80 years old. Anthropometric and biochemical characteristics were analyzed, and physical performance was evaluated. The statistical analysis used measures of central tendency and dispersion for descriptive data, Pearson correlations to demonstrate the initial associations between the variables, and canonical correlation (CC) to evaluate the relationship between the set of anthropometric adiposity indices and performance-related variables.

**RESULTS:**

The participants had a mean age of 58.57±8.21 years, a visceral adiposity index of 7.09±4.23, a body mass index of 29.20±4.94 kg/m^2^, and a conicity index of 1.33±0.07. The average handgrip strength was 25.06±4.89 kgf, gait speed was 1.07±0.23 m/s, and the mean Short Physical Performance Battery (SPPB) score was 10.83±1.36. The first canonical function presented the highest shared variance, CC, and redundancy index (cumulative percentage of variance, 82.52; Wilks’ lambda, 0.66; CC, 0.532; p<0.001). From the analysis of this canonical function, the conicity index (-0.59) displayed inverse correlations with handgrip strength (0.84) and the SPPB (0.68), as well as a direct correlation with gait speed (-0.43).

**CONCLUSIONS:**

In middle-aged and older women, there was an inverse relationship between the conicity index and muscle strength and power, while a direct relationship was found between the same index and gait speed.

## INTRODUCTION

Aging is responsible for several changes in the musculoskeletal system [1,2]. These changes include an increase in body adiposity and a reduction in muscle size with a consequent decrease in muscle strength, which are directly related to diminish physical function in older people [3,4].

Women generally have more fat mass than men, especially in later life due to the consequences of menopause and because they are less physically active [4-6]. Thus, they tend to have a higher prevalence of obesity and limitations in physical function [7,8].

Physical function is a term used to describe the ability of an individual to perform tasks of daily living [8]. It can be assessed by objective measures such as gait speed, the sit-and-stand test, and the Short Physical Performance Battery (SPPB) [9,10]. Additionally, physical function is predictive of important age-related outcomes, being a significant risk factor for disability, frailty, and mortality in the elderly [11,12].

The maintenance of physical function is directly related to successful aging [13]. Knowing the factors associated with the presence of functional limitations is extremely important, especially in women, since they have lower scores in physical performance indices than men in the same age group [12,14].

Regarding adiposity, the most commonly used measures for its assessment are the classic and well-known measures, such as the body mass index (BMI) and the waist-to-hip ratio (WHR) [15,16]. However, other indices have become prominent in recent years, such as the conicity index (CI), body adiposity index (BAI), and visceral adiposity index (VAI) [17,18]. In general, these indices have better differentiated visceral adiposity and overall adiposity, for which reason they could be considered more suitable measures of body adiposity. For instance, the BAI has been found to be more sensitive for identifying and classifying obesity than the BMI [19]. However, the current literature still lacks sufficient studies on the relationship between physical performance and these recently used adiposity indices.

Furthermore, previous studies have only demonstrated isolated relationships between each performance measure and each adiposity measure [1,20-22]. No studies have yet simultaneously analyzed the influence of a group of anthropometric indices of adiposity and a group of physical performance variables. Therefore, our intention was to explore multiple relationships between different indices and measures of functionality, indicating possible paths for future research to follow.

In the above context, the aim of the present study was to analyze the influence of anthropometric indices of adiposity on the physical performance of middle-aged and elderly women by canonical correlation (CC) analysis.

## MATERIALS AND METHODS

This cross-sectional, observational, and analytical study was carried out in 2 cities in the northeast of Brazil (Parnamirim and Santa Cruz), located in the state of Rio Grande do Norte. Northeast Brazil is one of the regions of the country with the highest degree of inequity, and the Rio Grande do Norte state occupies the 16th place in the development ranking among the 27 Brazilian states, with a human development index (HDI) of 0.684, which is considered medium in the HDI classification system. Most of the population is considered lower-middle class and vulnerable to poverty [23].

### Population and sample size

The study population consisted of women aged 40 years to 80 years. To be eligible for the study, participants were required to meet the following inclusion criteria: to be clinically healthy at the time of the interview, not to have undergone bilateral oophorectomy or hysterectomy, and not to have neurological diseases or other conditions that could compromise any stage of data assessment. In both Parnamirim and Santa Cruz, data collection was performed in Basic Health Units.

The sample was created by convenience after the project was advertised in Basic Health Units in all neighborhoods and community centers around the city. After the initial contact, the subjects were invited to go to the evaluation site, and after meeting the eligibility criteria, they were evaluated. In the age group selected for the study, 413 participants were initially recruited, of whom 45 were excluded because they had incomplete data related to biochemical tests and/or physical performance, totaling a final sample of 368 women.

### Procedures

The participants were evaluated by trained physical therapists using standardized protocols, while the blood samples were collected by trained nursing technicians and analyzed by specialized laboratory professionals. Data were collected on anthropometric indices, physical performance, biochemical parameters, sociodemographic characteristics, and physical activity, as described below.

### Anthropometric indices

#### Body mass index

BMI (kg/m^2^) was calculated from the measurement of height (m) and weight (kg) (World Health Organization, 2010). Weight (kg) was measured using a Wiso digital scale, model W903 (Wiso Tecnologia Esportiva, São José, Brazil). Height (m) was recorded using a Welmy stadiometer (Welmy Balanças, São Paulo, Brazil).

#### Waist-to-hip ratio

For waist circumference (WC; cm) and hip circumference (HC; cm) measurements, a fiberglass measuring tape with 1-mm divisions was used. WC was measured at the midpoint between the iliac crest and the last rib, and HC was measured at the most prominent area of the buttocks [16]. For the WHR calculation, the WC measurement was divided by HC.

#### Conicity index

The CI was calculated using the equation (CI= WC (m)/[0.109×√(weight (kg)/height (m))]), where 0.109 is a constant that results from converting units of volume and mass into units of length [20].

#### Visceral adiposity index and body adiposity index

Measurements were calculated according to previous studies found in the literature [17,18,22,24]. Triglycerides (TG) and high-density lipoprotein (HDL) cholesterol levels are expressed in mmol/L.

VAI: [WC (cm)/36.58+(1.89× BMI)]× (TG/0.81)× (1.52/HDL) BAI: (HC (cm)/height (m)^1.5^)−18

### Physical performance

Handgrip strength was obtained with a JAMAR portable dynamometer on the dominant hand (Ottoboni, Rio de Janeiro, Brazil). Each participant was seated with the shoulder fully adducted, in neutral rotation, and with the elbow flexed at 90° with the forearm and wrist in neutral positions [25,26]. Sustained contractions of 5 seconds were requested, with a 1-minute interval between measurements, and the arithmetic mean of 3 consecutive measurements was considered [27].

To evaluate physical performance, the SPPB was used in a version validated and adapted for the Portuguese language, which presented 3 subdivisions: standing balance, gait speed, and lower limb strength [28,29].

Balance was evaluated in 3 different positions for 10 seconds. Gait speed was evaluated with participants’ usual step for 4 meters, in which the shortest time of 2 attempts was recorded. Finally, to analyze lower limb strength, the sit-to-stand test was performed from a chair 5 times at maximum speed.

The total score of the SPPB is obtained by summing the 3 subdivisions, each of which has a maximum score of 4 points. Thus, the value can range from 0 (worst performance) to 12 (best performance) [30]. In this study, we used not only the total score of the SPPB, but also the values of the gait speed and the sit-to-stand test.

### Biochemical parameters

To calculate the VAI, it was necessary to analyze TG and HDL measurements. In this sense, the women were instructed to attend the Hospital Maternidade Divino Amor (Parnamirim) or the Hospital Universitário Ana Bezerra (Santa Cruz), according to the municipality they lived in, on a day and time previously scheduled, after a 12-hour fast, when blood samples were collected by trained nurse technicians. The levels of the biochemical parameters used, TG and HDL, were analyzed using the calorimetric enzymatic method by specialized laboratory technicians.

### Covariates

All covariates in this study were collected by reporting from the participants using a structured questionnaire. Marital status was categorized as married or unmarried. Education was categorized into less than basic education (up to 7 years), between basic and secondary education (more than 7 and less than 11 years), and secondary education or more (11 years or more). Family income was categorized as less than 3 times the minimum wage and 3 times the minimum wage or higher, according to the Brazilian minimum wage at the time of the survey [31]. Participants declared their race as White, Brown, or Black [32].

In addition, data regarding the practice of physical activity were also collected by self-reporting. The participants were asked whether they participated in sports, exercise, or other physical activities at least 3 times a week and for 30 minutes or more each time, with responses of “yes” or “no” [27].

### Statistical analysis

The statistical analyses were performed using SPSS version 20.0 (IBM Corp., Armonk, NY, USA). Initially, data normality was verified using the Kolmogorov-Smirnov test. Descriptive statistics were used, with measures of central tendency (arithmetic mean) and dispersion (standard deviation) for quantitative variables. Absolute and relative frequencies were used for categorical variables.

Pearson correlation analysis was performed to demonstrate the initial associations between the anthropometric indices and the physical performance variables. CC analysis was conducted to evaluate the relationship between the set of anthropometric indices of adiposity and the set of variables that constituted physical performance. This statistical technique is a type of multivariate analysis that allows the analysis of inter-relationships between groups of dependent and independent variables. The technique aims to determine linear combinations between groups in order to maximize the correlation between these combinations [33].

The group of dependent variables was formed by the physical performance variables: handgrip strength, gait speed, sit-to-stand test, and total SPPB score. The group of independent variables was composed of the anthropometric indices of adiposity.

The canonical loadings were derived from the CC analyses that provide the correlation between the original variables and the canonical variables, while Wilks’ lambda identified the significance of the canonical roots. Finally, the redundancy index, which reflects the ability of the set of independent variables to explain the variation in dependent variables, was used to explain the influence of anthropometric indices on physical performance.

For the selection of the canonical functions, the criterion of statistical significance of the function with p-value < 0.05 was established. The canonical loading value that defined the variables to be analyzed within each function was established as ± 0.40 [34].

Finally, during the analysis we evaluated multicollinearity within each group of variables according to the Montgomery et al. criterion [35].

### Ethics statement

This study received ethics approval by the Ethics and Research Committee of the Universidade Federal do Rio Grande do Norte (approval No. 1.875.802). All procedures in this study were in accordance with the code of ethics of the World Medical Association (Declaration of Helsinki). All participants were informed of the objectives and procedures of the research at first contact, and informed consent was obtained.

## RESULTS

The mean age of the participants was 58.57± 8.21 years. Regarding education, 194 (52.7%) had less than a basic education and 251 (68.2%) had an income lower than 3 times the minimum wage. The other characteristics of the sample, including the values for anthropometric indices and physical performance, are described in Table 1.

Table 2 shows the Pearson correlation values between the anthropometric indices and physical performance. All indices showed statistically significant correlations with more than 1 variable of physical performance, except for BMI, which was only correlated with handgrip strength (r= 0.12; p= 0.02).

The results of the CC analysis between the anthropometric indices of adiposity and physical performance of the sample are presented in Table 3. The first 3 canonical functions showed statistical significance, being responsible for 97.54% of the shared variance between the 2 sets of variables. In Table 3, it is important to consider that function 1 showed a good estimate of the variance shared between the 2 sets of variables, in addition to a higher CC and redundancy index (cumulative variance, 82.52%; Wilks’ lambda, 0.66; CC, 0.532; p< 0.001; redundancy index, 28.3%).

Table 4 shows the relationship of the canonical functions between the set of independent variables and the set of dependent variables. In the first canonical function CI (-0.59) showed inverse correlations with handgrip strength (0.84) and SPPB (0.68), as well as a direct correlation with gait speed (-0.43).

Regarding the second canonical function, it was observed that VAI (-0.52) and CI (-0.53) correlated inversely only with SPPB (0.47). Finally, the third canonical function showed inverse correlations of BAI (0.63), BMI (0.84), WHR (0.67), and CI (0.43) with gait speed (-0.59) and with SPPB (-0.45), and a direct correlation only with the sit-to-stand time (0.52). This table also shows the values of variance and CC, which indicate how much each canonical function contributed to the shared variance between groups, showing that the correlations found in the first function were the main responsible ones (accounting for 82.52% of the variance). Regarding multicollinearity, the results for both the condition number (less than 100) and the variance inflation factor (less than 1), the results indicated that multicollinearity was weak.

## DISCUSSION

Our results point out that physical performance measures were related to different anthropometric indices of adiposity in middle-aged and older women. The use of CC as a multivariate technique allowed us to analyze the complexity of the inter-relationships between all indicators of physical performance and adiposity simultaneously.

No studies have yet examined the association between physical performance measures and adiposity indices via CC analysis. More specifically, our results point out that the CI was associated with physical performance measures such as handgrip strength, SPPB and gait speed.

The current literature has pointed out that the CI is related to cardiometabolic risk factors [21,36]. However, there is still a lack of studies on the associations between this index and measures of physical performance in middle-aged and elderly women. Nonetheless, the impact of adiposity assessed by traditional measures, such as BMI and WC, on physical performance has been demonstrated in women [37]. A study conducted among 8,411 men and women aged 48-92 years found that for every increase in 10 cm in WC, there was a 1-kg reduction in handgrip strength in women, corroborating the inverse relationship between the CI and grip strength in our study [37].

Furthermore, the results of a systematic review indicate that there is a negative relationship between fat mass and physical performance in both genders older than 60 years [38]. A cohort study of 1,076 men and women aged 57-70 years that aimed to analyze BMI, WC, and body composition (electrical bioimpedance) as predictors of physical performance concluded that higher measures of adiposity were related to worse physical performance 10 years later [39]. Another study demonstrated that in overweight and obese elderly individuals, higher WC values were related to poor physical performance on the SPPB [40].

Contradictorily, our results showed a direct relationship between the CI and gait speed. The current literature points out that the presence of obesity and/or increased fat mass is related to lower gait speed values [41]. Wennman et al. [42], in a study of Finnish men and women, assessed the impact of obesity on walking speed, using only BMI and WC as markers of body and abdominal adiposity, respectively, and concluded that obesity in midlife can accelerate the decline in functional capacity as measured by maximal walking speed, especially in women.

However, in the present study, a greater number of markers of adiposity and their relationships with physical performance were analyzed. Moreover, the CI uses the variables of weight, height, and WC, and therefore presents a higher sensitivity and specificity than other measures for the evaluation of abdominal fat [43].

Moreover, according to Brach et al. [44], older women who remained physically active had better physical function regardless of obesity, although it is important to note that low income and low education were identified as detrimental factors to the adoption of regular physical activity in old age [45]. Chudyk et al. [46] reported that individuals aged ≥ 60 years represented the least physically active age group. However, it has been suggested that a low socioeconomic level makes these individuals more likely to walk or use public transportation instead of driving, causing them to engage in a certain level of physical activity.

Therefore, considering the socioeconomic characteristics of the present study population, which was mostly composed of low-income and low-education women, it is possible that they were more likely to walk, and thus, be active. Thus, they might have been able to be physically active even without regular exercise, which could also help justify our findings regarding gait speed.

The second canonical function showed that the VAI and CI were directly related to measures of handgrip strength, gait speed and sit-up time, and inversely proportional to SPPB. In the third function, BMI, the BAI, and the WHR were inversely related to gait speed and SPPB, and directly related to the sit-to-stand test. Although both functions were significant, the explanatory power of these functions was low (as expressed by the low percentage of variance explained).

Although our results cannot enable causal inferences, the relationship between obesity and poor physical performance is plausible, since studies have demonstrated the impact of obesity on muscle quality, such as the association between the presence of intramuscular fat and a negative influence on physical function in elderly women [47].

Furthermore, the presence of adiposity is associated with a pro-inflammatory state, which in turn is also related to poor functional performance [48]. Additionally, fat mass could also affect physical performance through some other mechanism; for example, atherosclerosis could impair physical performance through reduced cardiovascular function [39].

Thus, our study presents interesting results, since it evaluates this relationship considering all the components of each dimension studied. Additionally, the results from this study demonstrate the importance of controlling obesity, as well as encouraging an active lifestyle, in order to reduce the decline in physical performance in middle-aged and elderly women.

This study has some limitations. The fact that the analyses were cross-sectional limits causal inferences. Additionally, the use of a convenience sample makes it difficult to generalize our results. In addition, the gold standard for measuring body adiposity (dualenergy X-ray absorptiometry) was not used. However, anthropometric indices of adiposity have good reliability and validity, and are easily accessible for clinical practice.

Despite the limitations, the strength of the study is the uniqueness of the multivariate analysis used, considering that no studies in the literature have yet addressed the relationship between anthropometric indices of adiposity and measures of physical performance using canonical analysis. Furthermore, all variables used were objective, valid, non-invasive, and low-cost. This allowed a probable early detection of changes in adiposity indices that may result in limitations in women’s physical performance.

The results showed that physical performance is associated with changes in body composition that occur with age, although it is recognized that a decrease in physical performance due to other health reasons can affect changes in body composition and vice versa. The present study demonstrated the possibility of new perspectives for multivariate analysis through CC in the study of relationships between various adiposity indices and different measures of physical performance. According to our findings, the CI showed inverse relationships with handgrip strength and SPPB, while a direct relationship was found between the CI and gait speed.

## Figures and Tables

**Table 1.** Descriptive analysis (n=368)

Variables	Mean±SD or n (%)
Age (yr)	58.57±8.21
Marital status	
Married	244 (66.3)
Unmarried	124 (33.7)
Education	
Less than basic education	194 (52.7)
Between basic and secondary	111 (30.2)
Secondary or more	63 (17.1)
Household income	
<3 times the minimum wage	251 (68.2)
≥3 times the minimum wage	117 (31.8)
Race	
White	122 (33.2)
Brown	224 (60.8)
Black	22 (6.0)
Physical activity	
Yes	131 (35.6)
No	237 (64.4)
VAI	7.09±4.23
BAI	37.78±5.25
BMI (kg/m^2^)	29.20±4.94
WHR	0.92±0.05
CI	1.33±0.07
Handgrip strength (kgf)	25.06±4.89
Gait speed (m∕s)	1.07±0.23
Sit-to-stand (s)	11.39±4.75
SPPB	10.83±1.36

SD, standard deviation; VAI, visceral adiposity index; BAI, body adiposity index; BMI, body mass index; WHR, waist-to-hip ratio; CI, conicity index; SPPB, Short Physical Performance Battery.

**Table 2.** Pearson correlations between anthropometric indexes and physical performance variables in middle-aged and elderly women

Variables	Handgrip strength	Gait speed	Sit-to-stand	SPPB total
VAI	-0.05	0.12	0.04	-0.13
p-value	0.330	0.010	0.390	0.010
BAI	-0.08	0.01	0.16	-0.15
p-value	0.100	0.740	0.003	0.003
BMI	0.12	-0.08	0.08	-0.02
p-value	0.020	0.130	0.110	0.680
WHR	-0.14	0.03	0.11	-0.20
p-value	0.007	0.530	0.030	<0.001
CI	-0.20	0.13	0.19	-0.31
p-value	<0.001	0.010	<0.001	<0.001

SPPB, Short Physical Performance Battery; VAI, visceral adiposity index; BAI, body adiposity index; BMI, body mass index; WHR, waist-to-hip ratio; CI, conicity index.

**Table 3.** Canonical correlation (CC) analysis between anthropometric indices of adiposity and physical performance variables in middle-aged and elderly women

Canonical function	Auto value	Percentage of variance explained	Cumulative percentage of variance explained	CC	Wilks’ lambda	F	p-value	Redundancy index (%)
1	0.3949	82.52	82.52	0.532	0.66	6.90	<0.001	28.3
2	0.0387	8.09	90.61	0.193	0.92	2.18	0.011	3.7
3	0.0332	6.93	97.54	0.179	0.96	2.34	0.030	3.2
4	0.0118	2.46	100.00	0.107	0.99	1.85	0.158	1.1

**Table 4.** Canonical correlations between anthropometric indices of adiposity and physical performance variables in middle-aged and elderly women

Variable	Canonical function		
	First load	Second load	Third load
Anthropometry			
VAI	-0.23	-0.52	-0.08
BAI	-0.22	-0.24	0.63
BMI	0.20	-0.32	0.84
WHR	-0.39	-0.13	0.67
CI	-0.59	-0.53	0.43
Physical performance			
Handgrip strength	0.84	-0.49	0.01
Gait speed	-0.43	-0.68	-0.59
Sit-to-stand	-0.38	-0.49	0.52
SPPB	0.68	0.47	-0.45
Percentage of variance explained	82.52	8.09	6.93
Canonical correlation	0.532	0.193	0.179

VAI, visceral adiposity index; BAI, body adiposity index; BMI, body mass index; WHR, waist-to-hip ratio; CI, conicity index; SPPB, Short Physical Performance Battery.
